# Nuclear Pore Mechanotransduction in Oncology: A Structural Axis of Vulnerability for Targeted Intervention

**DOI:** 10.1002/adma.74655

**Published:** 2026-08-17

**Authors:** Sílvio Terra Stefanello, Caren Rigon Mizdal, Aline Franzen da Silva, Yun Qian, Wei‐En Yuan, Victor Shahin

**Affiliations:** ^1^ Institute of Physiology II University of Münster Robert‐Koch‐Straße 27b Münster Germany; ^2^ Department of Orthopaedics Shanghai Sixth People’s Hospital Affiliated to Shanghai Jiao Tong University School of Medicine Shanghai China; ^3^ Shanghai Engineering Research Center for Orthopaedic Material Innovation and Tissue Regeneration Shanghai China; ^4^ Shanghai Frontiers Science Center of Drug Target Identification and Delivery School of Pharmaceutical Sciences, Shanghai Jiao Tong University 800 Dongchuan Road Shanghai P. R. China; ^5^ National Key Laboratory of Innovative Immunotherapy Inner Mongolia Research Institute of Shanghai Jiao Tong University Shanghai China

**Keywords:** cancer, drug delivery, nanomedicine, nuclear mechanics, nuclear pore complexes

## Abstract

Nuclear pore complexes (NPCs) are massive protein assemblies that gate the nuclear envelope to regulate nucleocytoplasmic transport. Beyond their canonical role as conduits, NPCs act as mechanosensitive regulators that translate cytoskeletal, extracellular, and lamina‐derived forces into transport decisions coupled to cellular state. In malignancy, NPCs undergo extensive remodeling—marked by protein overexpression, hyperpermeability, and compromised scaffold integrity. While these alterations drive invasion and transcriptional plasticity, they simultaneously expose a lethal mechanical vulnerability; transformed cells become critically dependent on this altered NPC state. We propose the “nuclear mechanostat” as a testable conceptual framework in which NPC barrier stringency and nuclear mechanics are reciprocally coupled. Through the lenses of dilation, elastic deformation, and structural memory, we synthesize existing evidence and develop the hypothesis that pharmacological disruption of the permeability barrier or scaffold architecture erodes nuclear resilience. Because cancer cells operate near a mechanical failure threshold, targeting these conserved mechanotransducers reveals a pan‐cancer axis of vulnerability, opening new frontiers for precision oncology.

## Graphical abstract

Cancer‐associated NPC remodeling creates a high‐flux, low‐stringency nuclear state that supports malignant adaptation but increases mechanical fragility. Targeting the FG‐barrier or NPC scaffold may drive mechanostat failure, envelope rupture, DNA damage, and loss of nuclear integrity.

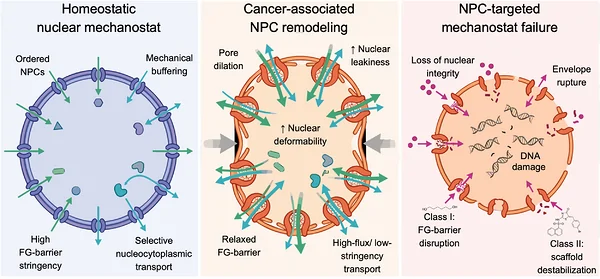

## 1 Introduction

Nuclear pore complexes (NPCs) are central orchestrators of cellular physiology, regulating gene expression, lineage specification, and the nuclear response to mechanical and metabolic stress. These processes, fundamental to cell identity and viability, are systematically hijacked by cancer cells to drive malignant progression. Indeed, the canonical hallmarks of cancer—including uncontrolled proliferation, metabolic reprogramming, and invasive behavior—rely on a fundamental rewiring of nuclear architecture and transport dynamics [1, 2, 3, 4, 5, 6, 7, 8].

Emerging evidence suggests that NPCs function as mechanotransductive interfaces that integrate cytoskeletal tension, extracellular matrix stiffness, and lamina‐derived forces [9, 10]. Rather than serving as passive conduits, NPCs appear to couple nuclear deformation with dynamic changes in transport selectivity and transcriptional adaptation [9, 11, 12, 13, 14, 15, 16, 17]. This concept is evolving, but accumulating data points to a role for NPCs in sensing and integrating mechanical cues, including force‐dependent changes in NPC permeability, pore dilation, and nuclear transport [9, 10, 11, 12, 13, 14, 15, 16, 17, 18, 19]. Throughout this Perspective, we distinguish between experimentally established observations and our proposed mechanistic model. Established evidence includes NPC structural plasticity, force‐dependent changes in pore architecture, cancer‐associated nucleoporin dysregulation, altered nuclear permeability, and lamina‐dependent nuclear mechanics. Building on these data, we propose the nuclear mechanostat as an integrative framework that links NPC barrier function to nuclear mechanical resilience in cancer.

In the high‐stress landscape of the tumor microenvironment, these forces converge on the nuclear envelope to reprogram NPC behavior. This remodeling—marked by the overexpression and structural reconfiguration of NPC proteins—represents a pathophysiological adaptation that reinforces the malignant phenotype rather than a mere byproduct of transformation [3, 6, 20, 21].

Direct evidence now supports the mechanical plasticity of the NPC. Atomic force microscopy (AFM) has demonstrated that NPCs undergo reversible dilation and constriction in response to physical perturbations, exhibiting remarkable elasticity and structural memory [18, 19, 22, 23]. These observations are complemented by studies showing that NPCs dynamically modulate nuclear permeability and fluid exchange in response to osmotic stress [10, 12].

These findings motivate our nuclear mechanostat model: a force‐responsive conceptual framework in which NPC barrier stringency and nuclear mechanics are proposed to be reciprocally coupled. We have previously demonstrated that the nucleocytoplasmic permeability barrier is not just a filter, but a primary mechanical determinant; its modulation directly recalibrates nuclear compliance, establishing the barrier as a second essential component of the mechanostat alongside the structural scaffold [9]. These studies support the view that NPC barrier stringency is not only a biochemical property of FG‐nucleoporins, but also a mechanically modulated feature of the nuclear envelope [16, 17, 24, 25, 26, 27, 28].

In this Perspective, we examine NPCs as mechanically responsive nano‐gates linking nuclear transport to nuclear mechanics. We first summarize established evidence on NPC structure, permeability, and cancer‐associated remodeling. We then discuss how these observations motivate the proposed “nuclear mechanostat” framework, in which NPC barrier stringency and nuclear mechanical resilience are hypothesized to be coupled. Finally, we outline how this framework may guide future strategies to test NPC‐centered mechanical vulnerabilities in cancer. To make this distinction explicit, Table 1 separates established evidence from supported interpretation and testable hypotheses.

**TABLE 1:** Evidence level supporting the main claims of this Perspective.

Main claim	Evidence status	Supporting evidence
NPCs are dynamic transport gates composed of scaffold, FG‐repeat, cytoplasmic, nuclear basket, and membrane‐associated nucleoporins.	Established evidence	Structural, biochemical, and transport studies have defined NPC architecture and receptor‐mediated nucleocytoplasmic trafficking [21, 29, 30, 31, 32, 33, 34, 35, 36].
NPC architecture and permeability can change in response to mechanical and biochemical cues.	Established/emerging evidence	Published studies show NPC dilation, deformation, altered FG‐barrier behavior, and mechanosensitive changes in nuclear transport [9, 10, 11, 12, 13, 14, 15, 17, 18, 19].
Many cancers show altered nucleoporin expression and increased reliance on nuclear transport machinery.	Established evidence	Cancer studies report dysregulation of individual nucleoporins, CRM1/XPO1 overexpression, altered import/export signaling, and changes in NPC number [3, 21, 37, 38, 39, 40, 41, 42, 43].
Cancer‐associated NPC remodeling can increase transport capacity while reducing barrier fidelity.	Supported interpretation	This interpretation is based on evidence of increased NPC abundance or transport activity, altered FG‐Nup modification, and enhanced nucleocytoplasmic flux in malignant contexts [7, 41, 42, 43, 44].
NPCs are mechanically coupled to the lamina, chromatin, and cytoskeletal force transmission.	Established/emerging evidence	Studies link Nup153–Lamin A/C interactions, nuclear envelope mechanics, chromatin organization, and force‐dependent nuclear deformation [16, 17, 24, 25, 26, 27, 28].
The “nuclear mechanostat” couples NPC barrier stringency to nuclear mechanical resilience.	Proposed framework	This is an integrative model built from established and emerging evidence on NPC mechanics, lamina mechanics, permeability regulation, and cancer‐associated nuclear fragility [7, 9].
Cancer cells may be selectively vulnerable to perturbation of NPC physiology.	Supported concept; context‐dependent	Inhibition of NPC formation has been shown to selectively kill cancer cells while normal cells undergo reversible arrest. Whether this extends to mechanical NPC‐targeting strategies requires direct validation [29, 44, 45].
Class I and Class II NPC‐targeting compounds may collapse mechanically stressed cancer nuclei.	Testable prediction	This is a proposed therapeutic extension of the mechanostat model and requires matched malignant versus non‐malignant testing to define selectivity and therapeutic window [7, 8, 9, 22, 46, 47, 48, 49].

## 2 The Nuclear Envelope as a Mechanosensitive Transport Hub in Oncology

### 2.1 Structural and Mechanical Organization of the Nuclear Pore Complex

NPCs are the master regulators of nucleocytoplasmic traffic, coordinating the genomic access and signal transduction pathways that define cellular identity. While essential for development and tissue homeostasis [29], this transport machinery is systematically hijacked during oncogenesis to support the aggressive metabolic and proliferative demands of the malignant phenotype [3, 30, 45].

Beyond its canonical transport role, the NPC is mechanically embedded in the nuclear envelope. Scaffold nucleoporins provide structural support and elasticity, FG‐repeat nucleoporins form a deformable permeability barrier, and nuclear basket and membrane‐associated components connect the NPC to lamins, chromatin, and nuclear envelope force‐transmission systems.

The NPC is a massive, highly organized assembly of approximately 30 different proteins, termed nucleoporins (Nups), which function as an integrated structural and mechanical system.

As illustrated in Figure 1, Nups are organized into three functionally interconnected modules [30]:

1. *The structural scaffold*: Accounting for roughly two‐thirds of the NPC mass, scaffold Nups (e.g., the Y‐complex: Nup107, Nup133, Nup160) utilize rigid α‐solenoid and β‐propeller domains to form a load‐bearing framework. These proteins organize into concentric rings that stabilize the nuclear envelope's high‐curvature pores, where the inner and outer nuclear membranes fuse to form a singular gateway. By lining this fusion point, they provide the mechanical chassis required to withstand cytoskeletal tension that would otherwise deform or collapse the nuclear openings [31].
2. *The permeability barrier (FG‐Nups)*: Approximately one‐third of the NPC is composed of intrinsically disordered proteins (IDPs) rich in phenylalanine: glycine (FG) repeats (Figure 1A). Tethered to the scaffold, FG‐Nups behave as a dynamic biopolymer condensate that projects into the central channel. This disordered assembly creates a selective filter, functioning as a tunable barrier that regulates molecular flux through the pore [18, 22, 32, 50, 51, 52]. Current biophysical models describe this barrier a selective hydrogel mesh [52], an entropic polymer brush [53], or a heterogenous “forest” of transport zones [54]. Unlike static filters, these disordered domains are highly responsive to their chemical and physical environment. Importantly, certain FG‐Nups exhibit rapid turnover or “shuttling” behavior [34], allowing the NPC to recalibrate its transport kinetics in response to acute stress [29, 35].
3. *Transmembrane anchors*: They bridge the nuclear envelope bilayer, physically anchoring the scaffold rings to both the lipid membrane and the underlying nuclear lamina [35, 36]. This architecture creates a continuous mechanical conduit, essential for transducing environmental mechanical forces directly from the cytoplasm into the nuclear interior. This mechanical coupling is not restricted to direct NPC–lamina contacts. NPCs operate within a broader nuclear‐envelope network that includes lamins, chromatin‐associated proteins, and the LINC complex. SUN1 and SUN2, together with nesprins, connect the nuclear interior to cytoskeletal forces and may influence NPC positioning, nuclear‐envelope organization, and force transmission [16, 17, 20, 26, 55].

**FIGURE 1:**
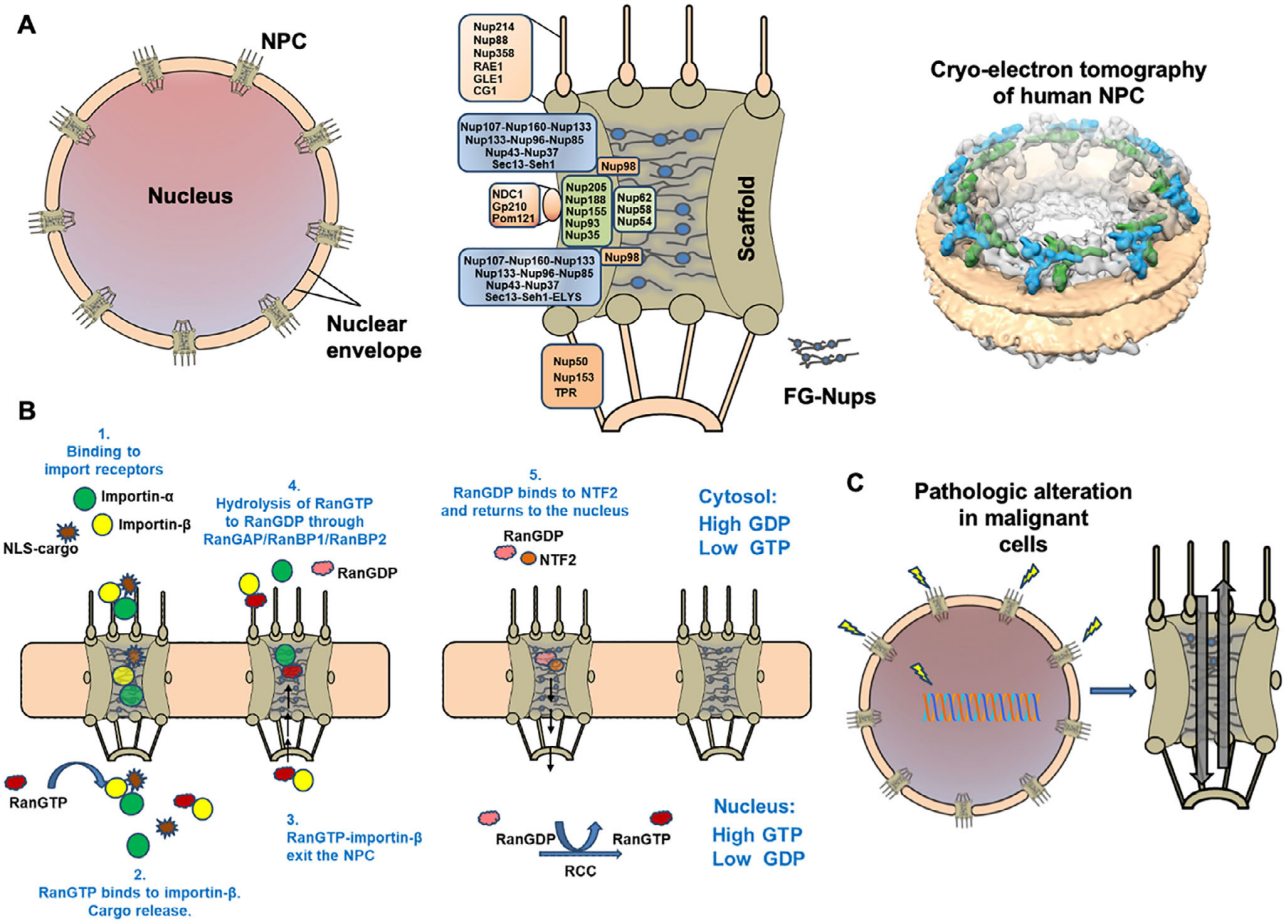
Structural and functional organization of the nuclear pore complex, transport modes, and cancer‐associated remodeling. (A) NPCs span the nuclear envelope and are composed of nucleoporins organized into scaffold, central‐channel, cytoplasmic, nuclear‐basket, and membrane‐associated modules. The scaffold includes Y‐complex assemblies that stabilize the pore membrane, while FG‐Nups form the selective permeability barrier [31]. (B) Receptor‐mediated nucleocytoplasmic transport occurs through importin/exportin‐dependent cargo recognition and RanGTP/RanGDP‐driven directionality. It is a major regulatory axis in cancer, where increased nucleocytoplasmic flux supports rapid proliferation, oncogenic signaling, and transcriptional adaptation [42, 62]. Passive diffusion may also become relaxed when FG‐Nup expression or biochemical properties, including O‐GlcNAcylation, are altered [63]. The relative contribution of active vs. passive transport remodeling in cancer remains under investigation. (C) Proposed working model of cancer‐associated NPC remodeling. Altered nucleoporin expression, increased transport demand, changes in NPC abundance or organization, CRM1/XPO1‐dependent export bias, and reduced FG‐barrier fidelity are schematically integrated into a high‐flux but less stringent nucleocytoplasmic transport state. While individual components of this remodeling are supported by published evidence, the integrated “massive but leaky” state is presented here as a conceptual synthesis.

Transport through the NPC occurs via two distinct modes (Figure 1B). Passive diffusion permits small molecules (typically up to ∼40 kDa or ∼4 nm in diameter) to transit through the FG mesh without assistance [30]. Larger macromolecules are physically excluded due to steric hindrance and entropic repulsion. For molecules exceeding this threshold, receptor‐mediated transport is required [30]. This process involves soluble nuclear transport receptors (NTRs), such as importins and exportins, which recognize nuclear localization signals (NLS) or nuclear export signals (NES) on cargo proteins. These receptors bind their cargo and interact transiently with FG repeats, enabling rapid, selective translocation across the NPC barrier. The ability of NTRs to dissolve into the FG mesh and shuttle cargo across the pore is central to NPC function [35]. This distinction is particularly relevant in cancer, where receptor‐mediated transport is frequently amplified to sustain rapid proliferation, oncogenic transcriptional programs, and stress adaptation. At the same time, passive permeability may also be relaxed through altered FG‐Nup expression and biochemical remodeling, including changes in O‐GlcNAcylation. How active and passive transport routes are differentially remodeled across tumor types remains an active area of investigation [56, 57, 58, 59, 60].

In cancer, both transport modes—passive diffusion and receptor‐mediated translocation—are fundamentally dysregulated (Figure 1C). The overexpression of export factors like CRM1 [61], coupled with post‐translational modifications of key Nups (specifically O‐GlcNAcylation of Nup62 and Nup98) [56, 57, 58, 59, 60], dynamically reshapes NPC behavior to meet the demands of transformed cells [21, 56, 60]. These changes represent a strategic reprogramming of the intracellular structure, analogous to how cancer cells remodel the extracellular matrix and vasculature during tumor progression. Through these adaptations, NPCs evolve from passive conduits into active regulators of oncogenic signaling, stress response, and gene expression, providing a critical interface for therapeutic intervention. Thus, Figure 1C should be read as a schematic summary of the cancer‐associated changes developed further in Section 2.2: altered Nup expression, increased transport demand, CRM1/XPO1‐driven export bias, post‐translational FG‐Nup remodeling, and reduced barrier fidelity, as outlined in Table 1.

### 2.2 NPC Remodeling and Transport Dysregulation in Cancer Pathophysiology

In healthy cells, NPCs operate as high‐fidelity checkpoints that balance selective flux with compartmental integrity. In malignancy, this equilibrium is systematically disrupted to support the biosynthetic and transcriptional “overdrive” required for rapid proliferation (Figure 1C) [3]. Cancer cells frequently exhibit a “massive but leaky” phenotype: a paradoxical state where the overexpression of specific Nups coincides with a catastrophic reduction in barrier stringency. This transport upregulation is not a byproduct of transformation but a primary requirement to accommodate the massive metabolic demands of the malignant cell (Figure 1C) [64].

The expression of specific nucleoporins is significantly elevated across a broad spectrum of malignancies, where they function beyond simple gatekeeping as transcriptional regulators and signaling modulators [31, 37, 38, 65]. In several malignancies, this proteomic remodeling correlates with poor prognosis, metastatic potential, and altered therapy response [3, 21, 37, 38, 39, 40, 41, 42, 43]. In parallel, cancer cells upregulate nuclear export receptors such as CRM1 (XPO1) [39, 60, 61, 64], creating a profound export bias that clears tumor suppressors and stress mediators from the nuclear compartment. Although CRM1/XPO1 inhibitors have entered clinical use, their activity as monotherapies is often limited by toxicity, resistance, and pathway redundancy, suggesting that targeting a single export route may be insufficient [3, 21, 37, 38, 39, 40, 41, 42, 43]. Instead, NPC remodeling must be understood as a coordinated, system‐wide mechanical and chemical adaptation.

We have previously demonstrated that NPCs possess exceptional structural elasticity and mechanical memory [22]. Using AFM and biochemical assays, we showed that NPCs can undergo reversible dilation to accommodate macromolecules far exceeding the classical diffusion cutoff—including large protein complexes and therapeutic nanoparticles [50, 66, 67]. This adaptability reframes NPCs as force‐sensitive valves capable of recalibrating their “mesh size” in response to cellular stress.

In cancer, this dynamic behavior is hijacked to facilitate the rapid translocation of oncogenic regulators [3]. Whether through Nup overexpression or structural reconfiguration, NPC remodeling lowers the energetic barrier to transport and promotes the breakdown of nuclear compartmentalization. O‐GlcNAcylation of Nup62 and Nup98 has been implicated as one important contributor to reduced NPC barrier stringency, which acts as a biochemical wedge to weaken the exclusion barrier [56, 68].

Crucially, NPCs do not operate in isolation. Their function is coupled to the mechanical state of the nuclear envelope, lamina, chromatin, and LINC‐dependent cytoskeletal force transmission. Independent studies have shown that nuclear deformation, lamina organization, pore dilation, and mechanosensitive transport can alter nuclear import/export behavior. Building on this evidence, we use the term “nuclear mechanostat” to describe a proposed framework in which NPC barrier stringency and nuclear mechanical resilience are functionally linked. This concept is summarized in Table 1 and developed schematically in Figure 3 [16, 17, 69].

### 2.3 Multi‐Scale Filtration: Remodeling of Tissue and Cellular Infrastructure in Cancer

Selective permeability is a cornerstone of physiological regulation. In renal physiology, the glomerular filtration barrier governs molecular passage based on size, charge, and shape, balancing hydrostatic pressure and osmotic gradients to maintain compartmental integrity. This multilayered system, composed of fenestrated endothelium, basement membrane, and podocyte slit diaphragms, functions as a dynamic filter. Pathological states such as proteinuria arise when this balance is disrupted, allowing macromolecules to leak into the urine and signaling systemic dysfunction.

As a conceptual analogy, we propose a filtration paradigm for nuclear transport that draws mechanistic parallels between the glomerular ultrafiltration barrier and the NPC. We emphasize that this Starling‐like description is used here as a conceptual analogy, not as an established consensus model of nucleocytoplasmic transport. It is placed alongside complementary models of NPC selectivity, including selective‐phase/hydrogel, polymer‐brush, virtual‐gate, and transport‐zone models [33, 38, 53, 54, 70].

Like the renal glomerulus, NPCs resolve the fundamental conflict between high‐volume flux and stringent selectivity across a compartmental boundary. This permeability is governed not by static apertures but by dynamic, soft‐matter domains of FG‐Nups that modulate the interface in response to mechanical and physiological cues [12, 14, 22, 32, 50]. Specifically, these IDPs form a biopolymer condensate that sustains a selective barrier by continuously reshaping transient voids [71]. FG‐Nups thus serve as functional analogs to podocyte slit diaphragm proteins, such as nephrin and podocin [72]; both systems leverage the biophysical properties of IDPs to maintain a selective interface. These IDP‐rich systems utilize a tunable, flexible meshwork to sustain permeability barriers under varying physiological pressures. Key shared components include a structural core of scaffold proteins (e.g., nephrin/Neph1) that anchor the disordered domains, and specialized adapter proteins (e.g., podocin) that modulate the meshwork's density and signaling response, mirroring the organizational logic of the NPC. By reframing the NPC as a dynamic biopolymer filter, we provide a unified biophysical basis for understanding how nuclear gating is coupled to cellular mechanostates.

NPC selectivity is governed by a complex interplay of size‐exclusion, molecular charge, and hydrophobicity [51, 52, 73, 74, 75], aligning with the principles of soft‐matter physics [71]. The transport of cargo through the NPC is governed by two synergistic properties: hydrophobicity and electrostatic charge. Hydrophobic interactions with the FG‐Nup meshwork allow transport receptors to partition into the pore for rapid passage. Simultaneously, the NPC creates a positively charged internal environment that acts as a selective filter. Rather than simply being excluded, positively charged molecules are repelled by this environment, while transport receptors utilize a high net negative charge to navigate the pore via electrostatic attraction, which lowers the energy barrier for translocation [74, 76]. These findings reinforce the view of the NPC as a chemically and mechanically responsive biopolymer condensate. Critical post‐translational modifications, particularly the O‐GlcNAcylation of Nup62 and Nup98, further modulate this filter [77].

Elevated O‐GlcNAc levels significantly accelerate bidirectional transport by weakening cohesive FG‐interactions, thereby enhancing nonspecific permeability and nearly doubling mRNA export efficiency [63].

In malignancy, altered metabolic flux through the hexosamine biosynthetic pathway upregulates O‐GlcNAcylation to fundamentally reshape the nuclear boundary, a process integrated with the structural remodeling of the nuclear lamina and peripheral chromatin [78]. We propose that this modification acts as a biochemical wedge; it stabilizes Nup62 and Nup98 against proteasomal degradation while simultaneously loosening their cohesive FG‐mesh. We refer to this remodeled NPC state as “massive but leaky,” denoting the experimentally observed coexistence of elevated transport capacity with reduced barrier fidelity. This phenomenon represents a form of nuclear hyperpermeability analogous to the bloated, fragile vasculature of tumor‐induced angiogenesis. Just as leaky vessels facilitate systemic dissemination, this compromised boundary accelerates the bidirectional exchange of oncogenic signaling effectors and mRNA. By lowering the energetic barrier to transport and disrupting peripheral gene silencing, this “nuclear leakiness” provides the essential substrate for transcriptional plasticity, enabling the rapid genomic rewiring required for invasion and therapeutic resistance [79]. This analogy is supported by our prior work combining AFM, confocal imaging, and biochemical assays to show that NPCs exhibit pressure‐sensitive behavior, reversible dilation, and dynamic gating [9, 22, 50], properties long recognized in renal filtration but not previously attributed to nuclear transport. The resulting ultrafiltration model of nuclear mechanics reframes NPCs as force‐sensitive valves embedded within a pressurized envelope, modulating permeability in response to mechanical load and biochemical state. This behavior is particularly relevant in cancer, where transformed cells are subject to elevated mechanical stress and disrupted tissue architecture.

Mechanical stress is not merely a consequence of tumor growth but a primary driver of nuclear reprogramming. The tumor microenvironment—defined by increased matrix stiffness, compressive forces, and disrupted cytoskeletal architecture—converges on the nuclear envelope to modulate pore architecture. We propose that under these conditions, FG‐Nup domains undergo force‐induced remodeling, permitting transient dilation and enhanced nuclear access. This mechanosensitive shift allows cancer cells to rapidly import transcriptional regulators and export stress mediators, facilitating dynamic adaptation to environmental demands. In this state, the nucleus—once a strictly buffered compartment—is transformed into a vulnerable interface, exposed to cytoplasmic fluctuations and unable to sustain the transport fidelity required for homeostatic gene expression.

This filtration paradigm reframes NPC remodeling not as a passive byproduct of transformation, but as an active mechanoadaptation to oncogenic stress. While this plasticity supports invasive behavior and transcriptional reprogramming, it simultaneously introduces a lethal fragility: the loss of barrier stringency heightens susceptibility to nuclear rupture, DNA damage, and chronic cGAS‐STING activation [80]. These vulnerabilities are acutely amplified in the high‐stress tumor microenvironment, where nuclear filtration is most compromised. Furthermore, the overexpression of export receptors such as CRM1 exacerbates this imbalance; by shifting the macromolecular equilibrium toward the cytoplasm, CRM1‐mediated “export overload” erodes nuclear resilience, mirroring the convective loss observed in leaky tumor vasculature. This model extends beyond simple selectivity, positioning NPCs as pressure‐sensitive valves that dictate cellular fate at the interface of tissue mechanics and nuclear homeostasis.

As illustrated in Figure 2, tumor‐induced remodeling of the extracellular matrix and neovasculature elevates interstitial fluid pressure (Δ*P*), driving abnormal macromolecular distribution. We propose a parallel Starling‐like mechanism at the nuclear envelope: increased NPC permeability and the resulting imbalance between active import and export fundamentally alter the nuclear colloid‐osmotic pressure (ΔΠ), reshaping nucleocytoplasmic exchange. This analogy reinforces the concept that cancer reconfigures both extracellular and intracellular infrastructure to meet its metabolic demands. NPCs, like endothelial barriers, function as dynamic, mechano‐chemically tuned regulators of compartmental access that support malignant progression. By extending this filtration paradigm from renal physiology to nuclear transport, we identify a mechano‐transductive axis that links tissue‐scale stress to molecular‐scale selectivity. This model integrates principles from oncology, medical physiology, and soft‐matter physics to redefine the nucleus as a tissue‐responsive system. We will next explore how this axis is governed by a unified regulatory framework: the nuclear mechanostat.

**FIGURE 2:**
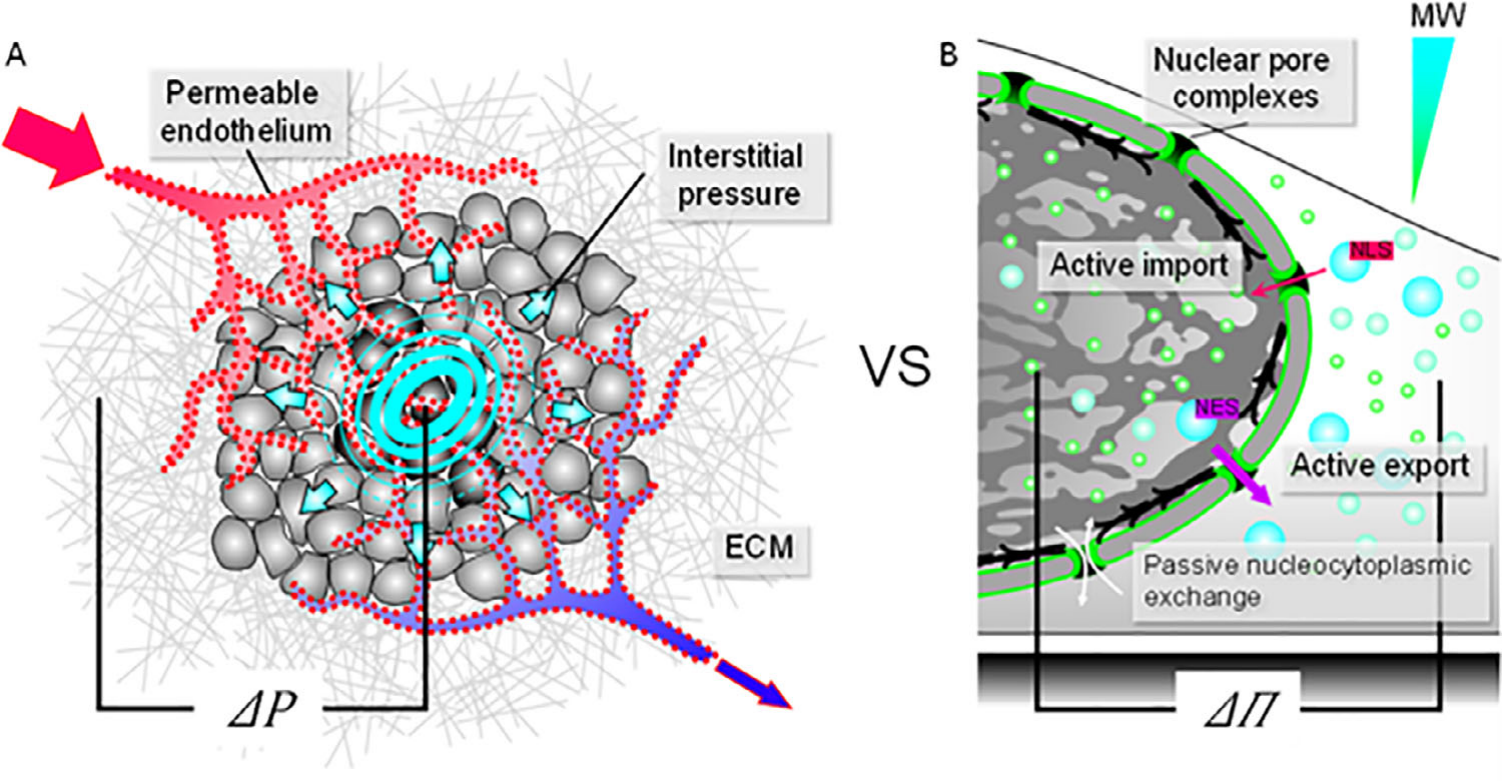
Parallelisms in multi‐scale filtration and infrastructure remodeling in cancer. (A) Tissue‐scale remodeling: Tumor progression involves extensive neovascularization and extracellular matrix (ECM) reorganization. Increased endothelial permeability combined with impaired lymphatic drainage leads to elevated interstitial fluid pressure (Δ*P*). This pressure gradient drives the extravasation of macromolecules and sustains a stiffened microenvironment, modulating mechanical stress propagation across the tissue. (B) Cellular‐scale remodeling: A parallel Starling‐like principle governs transport across the nuclear envelope. Upregulated NPC permeability and an imbalance in nucleocytoplasmic flux disrupt the colloid‐osmotic pressure gradient (ΔΠ). This shift alters macromolecular crowding, nuclear mechanostability, and transcriptional fidelity. Overall concept: Analogous to the “leaky” tumor vasculature, NPC remodeling represents a strategic reprogramming of cellular infrastructure to support oncogenic metabolic and transcriptional demands. Both systems transition from strictly regulated gates to permeability‐disrupted interfaces, creating a unique mechanical vulnerability that offers a targeted axis for precision oncology.

### 2.4 Mechanotransductive NPC Remodeling and the Nuclear Mechanostat

NPCs are integrated nodes within the force‐transmission network linking the cytoskeleton to the nuclear interior. This connection is anchored by a mechanosensing axis between Nup153 and Lamin A/C, which tethers the NPC basket to the underlying nuclear lamina [55]. Anchored to both the lamina and the pore membrane, NPCs directly experience and transduce mechanical stresses propagated via the LINC complex and the actomyosin cytoskeleton. These forces dictate NPC spatial distribution and gating kinetics, positioning the pore as a primary mechanosensitive regulator rather than a passive conduit. This view is supported by independent studies showing that nuclear deformation, lamina organization, LINC‐mediated force transmission, NPC dilation, and cargo mechanics can modulate nuclear transport and mechanotransductive signaling [13, 14, 15, 16, 17, 24, 27, 28].

We propose that NPC barrier stringency and nuclear mechanics are operatively coupled: barrier relaxation increases nuclear compliance, whereas reinforcement enhances mechanical stiffness. In this framework, the FG‐Nup permeability barrier functions as a biophysical buffer, generating the internal resistance necessary for the nucleus to adapt to deformation while preserving compartmental integrity. We define this proposed reciprocal relationship as the nuclear mechanostat—a rapid‐response regulatory system that integrates permeability control with mechanical adaptation (Figure 3) [9].

**FIGURE 3:**
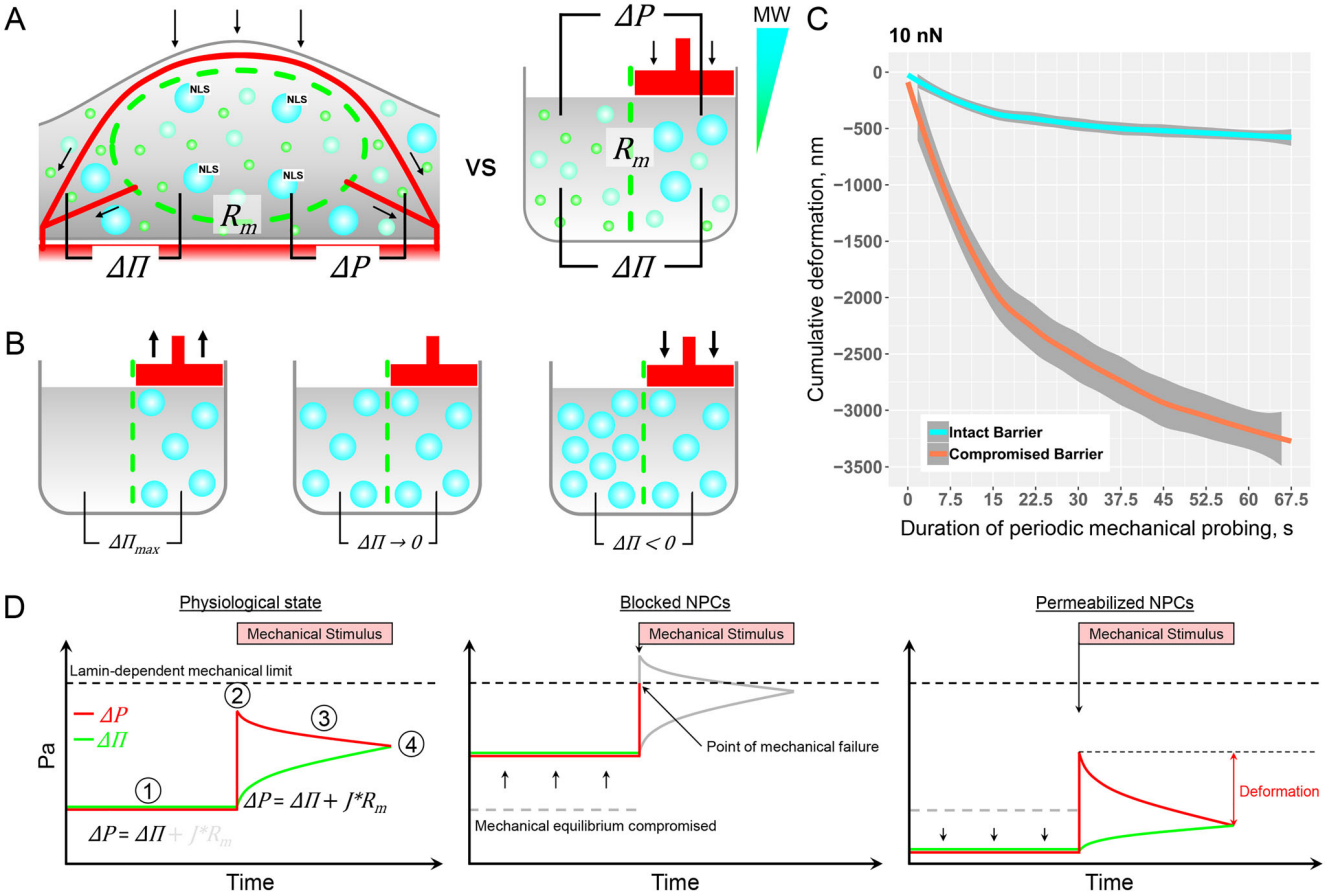
Conceptual ultrafiltration model of nuclear mechanics and its implications for transport fidelity and genomic instability. The schematic is redesigned for this Perspective and based on the ultrafiltration framework developed in our previous work [9]. (A and B) Theoretical framework: The nucleus is modeled as an ultrafiltration system comprising two compartments separated by a selective boundary with a dynamic molecular weight cut‐off. External mechanical forces generate hydrostatic pressure (Δ*P*) across the nuclear envelope, while the sequestration of large macromolecules creates a counteracting colloid‐osmotic pressure (ΔΠ). The balance of these Starling‐like forces dictates nuclear volume and structural stability. (C) NPC‐mediated compliance: Experimental data demonstrate that increased NPC permeability leads to greater nuclear deformability under identical mechanical loads. This confirms model predictions that the NPC serves as a tunable regulator of nuclear compliance. (D) Mechanostat failure and rupture: Under physiological conditions, colloid‐osmotic pressure acts as a mechanical buffer, absorbing external forces to maintain envelope integrity. Pathological NPC blockage raises baseline internal pressure, making even moderate mechanical loads sufficient to exceed the envelope's elastic limit. Conversely, malignant NPC permeabilization (the “leaky” state) prevents osmotic buildup, leading to excessive deformation, structural memory loss, and increased genomic instability through mechanical failure.

In cancer, we propose that the set point of this mechanostat is recalibrated. Disruption of the Nup153‐Lamin A axis—a hallmark of many malignancies—leads to “mechanostat failure” [55], where the balanced regulation of permeability and structural resilience is lost. This favors a high‐flux, low‐stiffness state that facilitates nuclear deformation during invasion while simultaneously resulting in the characteristic nuclear “leakiness” and heightened susceptibility to mechanical failure observed in transformed cells.

Based on the available evidence, we hypothesize that cancer cells exploit NPC mechanotransductive capacity through a two‐stage process of acute response and chronic adaptation. Acute mechanical stress‐such as that experienced during confined migration‐triggers rapid, tension‐gated NPC dilation, permitting the transient entry of large cargoes. We suggest that cancer cells subsequently stabilize and exploit this “open” state through chronic, multi‐layered remodeling: altered Nup expression profiles, post‐translational modifications (e.g., O‐GlcNAcylation), and scaffold reconfiguration. These changes collectively reduce barrier stringency and lower the energetic threshold for transport, thereby accelerating the bi‐directional exchange of oncogenic signaling effectors and mRNA.

This remodeling is tightly coupled to changes in overall nuclear architecture. Lamin A/C depletion‐a common feature of highly invasive tumors‐reduces intrinsic nuclear stiffness and enhances deformability, sensitizing NPCs to mechanical input transmitted via the LINC complex. Concurrently, chromatin decondensation near the nuclear periphery increases NPC accessibility and facilitates gene activation, while cytoskeletal tension modulates pore distribution and gating behavior. Together, these interdependent changes create a permissive mechanogenomic landscape for force‐driven transport activation and rapid phenotypic adaptation.

We further propose that the overexpression of export receptors, such as CRM1, exacerbates this transport imbalance. By shifting the macromolecular equilibrium toward the cytoplasm, CRM1‐mediated export fundamentally reduces the intranuclear colloid‐osmotic pressure (ΔΠ). This decrease in internal resistance erodes nuclear resilience, facilitating the extreme deformability and physical “squeezing” required for metastasis.

Recent studies confirm that this rewired system is exploited to facilitate nuclear import of oncogenic transcription factors such as, YAP/TAZ, NF‐κB, and c‐Myc, while promoting the export of stress mediators like HSPs and HMGB1 [46, 55, 63, 80, 81]. Critically, under extreme stress, this chronic state of NPC permeabilization can lead to catastrophic nuclear envelope rupture, uncontrolled nucleoctoplasmic exchange, and DNA damage.

Together, these findings support a model in which NPCs act as force‐sensitive valves embedded within a mechanically integrated nuclear envelope. By translating mechanical cues into transport decisions, they regulate transcriptional flux, stress adaptation, and genomic stability. In cancer, this mechanotransductive system is rewired to support malignant progression but also reveals a therapeutic vulnerability. In the next chapter, we examine how this force‐responsive transport axis is governed by the nuclear mechanostat, a dynamic framework that orchestrates stress‐adaptive transport control.

### 2.5 Transport Selectivity Under Mechanical Stress: A Therapeutic Vulnerability in Cancer

Nucleocytoplasmic transport is not merely biochemical; it is mechanically gated and governed by the nuclear mechanostat, which integrates energy landscapes, structural anisotropy, and dynamic force transmission to maintain nuclear homeostasis. Mechanical forces arising from extracellular matrix stiffness, cell–cell contacts, and cytoskeletal contractility modulate nuclear envelope tension and pore architecture, thereby reshaping transport selectivity. This view builds on a broader mechanotransduction literature showing that nuclear mechanics, transport, and genome regulation are physically coupled. Matrix stiffness and cytoskeletal tension regulate nuclear flattening and YAP/TAZ nuclear import; lamins and the LINC complex transmit cytoskeletal forces to the nuclear envelope; chromatin organization contributes to nuclear stiffness and deformability; and excessive deformation during confined migration can trigger nuclear envelope rupture, DNA damage, and cGAS–STING activation. Together with recent work on NPC dilation, permeability regulation, and cargo mechanoselectivity, these studies position the NPC within a larger nuclear mechanotransduction network rather than as an isolated transport channel [10, 13, 14, 15, 16, 17, 24, 25, 26, 27, 28, 55].

Recent studies reveal that NPC behavior spans a continuum of mechanical states. Cryo–electron tomography shows that NPCs dilate in metabolically active cells and constrict under osmotic or energetic stress [10]. Nuclear flattening under force enhances import of YAP by reducing mechanical resistance at the pore interface [14], establishing a biophysical model in which deformation lowers the free‐energy barrier for translocation.

Panagaki et al. [15] extended this paradigm by showing that cargo mechanics influence transport kinetics. Proteins with locally soft regions near nuclear localization sequences exhibit higher import rates, while stiff cargos face entropic and energetic penalties. This mechanoselectivity is mediated by Nup153, which integrates extrinsic force and intrinsic cargo deformability into transport decisions.

Viewed through a physicist's lens, NPCs resemble tunable energy barriers modulated by external stress and internal entropy. The permeability landscape is dynamic and is reshaped by mechanical inputs that alter pore geometry, scaffold elasticity, and FG‐domain cohesion. Transport becomes a probabilistic event governed by force‐dependent conformational transitions and cargo‐specific mechanical signatures.

In healthy cells, this mechanosensitive gating preserves nuclear fidelity by ensuring that transcriptional regulators and stress mediators are imported only under appropriate mechanical conditions. In cancer, this regulatory architecture is dismantled. Transformed cells exhibit elevated transport flux, reduced barrier stringency, and decoupling from mechanical feedback. NPCs operate as high‐throughput conduits, amplifying signaling noise and accelerating phenotypic plasticity.

This breakdown creates a paradoxical vulnerability. Cancer cells depend on unbuffered transport to sustain their malignant phenotype, yet this dependence renders them mechanically fragile. Envelope rupture, DNA damage, and transport overload are frequent in invasive tumors, reflecting the cost of abandoning mechano‐adaptive control.

By restoring mechanosensitive gating or selectively destabilizing it, we can reimpose transport fidelity and exploit the fragility of cancer's deregulated exchange machinery. NPCs thus emerge as therapeutic interfaces, not merely structural gateways but core actuators of the nuclear mechanostat. The next section explores how chemical modulators, nanoparticle‐based delivery systems, and scaffold‐disrupting agents can leverage this vulnerability, transforming transport imbalance into a precision‐guided axis of intervention.

### 2.6 Pharmacological Modulation of NPC Mechanics: A Precision Strategy for Intervention

The mechanical adaptability of NPCs is central to their role in cancer progression. Far from passive conduits, NPCs are dynamic, force‐responsive structures whose permeability and mechanical behavior are tightly coupled to nuclear mechanics. In transformed cells, this coupling is exploited to sustain elevated nucleocytoplasmic flux and the extreme deformability required for confined migration. However, this adaptive remodeling creates a profound therapeutic vulnerability: malignant cells become critically dependent on a “mechanically primed” NPC state. Targeting this interdependence may offer a mechanophysiologically guided intervention strategy designed to test whether collapse of the nuclear mechanostat can be therapeutically exploited.

More broadly, several lines of work support the idea that cancer cells can become unusually dependent on NPC function, nuclear transport capacity, and NPC biogenesis. For example, inhibition of NPC formation has been shown to selectively induce cancer cell death, whereas normal cells can undergo reversible cell‐cycle arrest [44].

Our group has developed two mechanistically distinct classes of compounds that disrupt NPC mechanics and transport fidelity (Figure 4). These agents, designated as *Class I and Class II inhibitors*, target the structural elements governing mechanical gating. Class I inhibitors destabilize the cohesive interactions within the permeability barrier, exacerbating “nuclear leakiness” to a point of catastrophic loss of transport control. *Class II inhibitors* target the structural scaffold, undermining the NPC's ability to withstand cytoskeletal tension and osmotic fluctuations.

**FIGURE 4:**
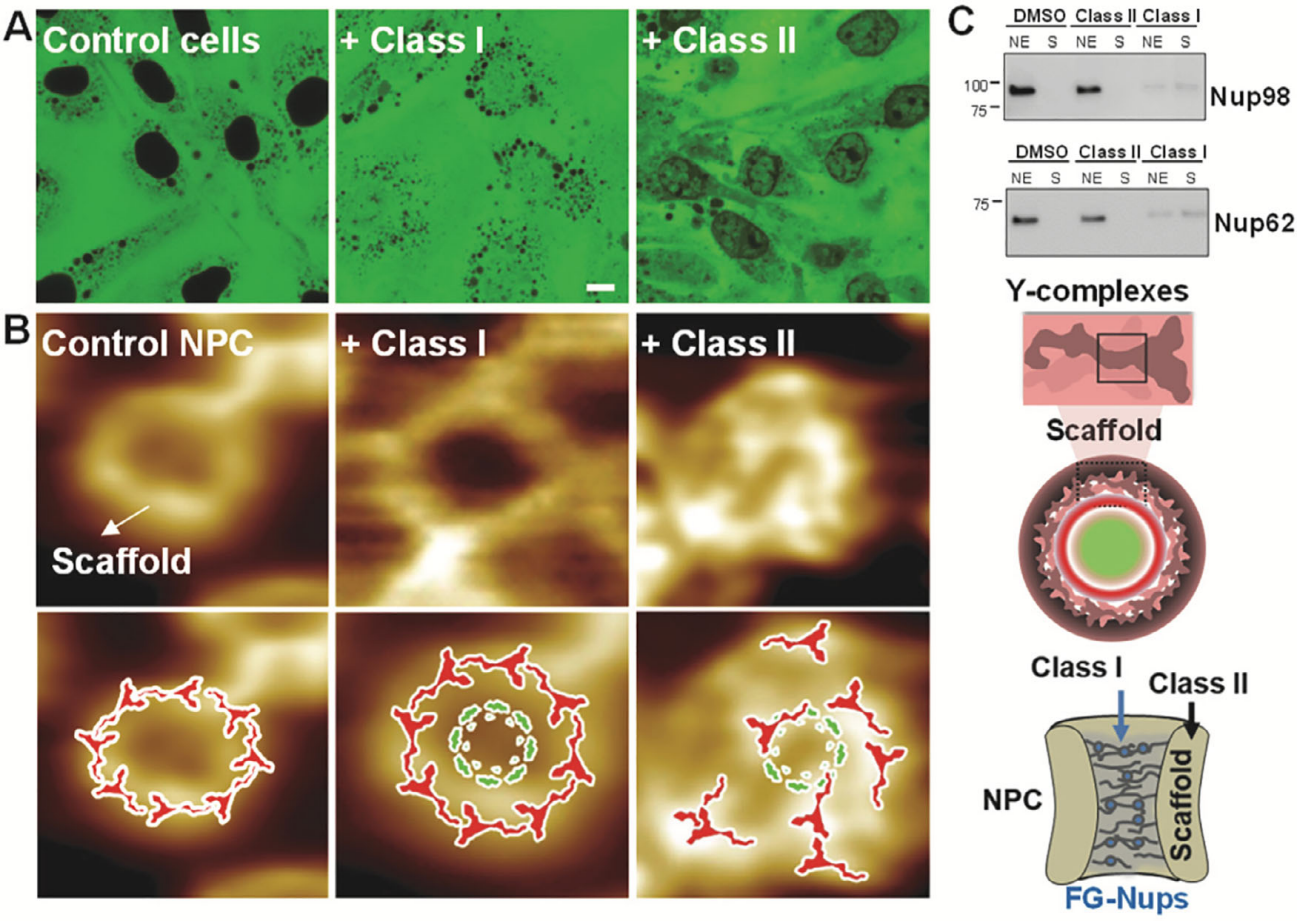
Assessment and pharmacological modulation of NPC permeability with Class I and II compounds. (A) Digitonin‐permeabilized cells retain an intact nucleocytoplasmic barrier, excluding 70 kDa fluorescent Dextran from the nucleus (left). Barrier disruption by Class I or II compounds permits nuclear entry of the tracer (middle, right). Scale bar: 10 µm [46]. (B) AFM reveals distinct mechanisms: Class I induces NPC channel dilation [81]; Class II disrupts scaffold ring integrity [46]. Scale bar: 60 nm. (C) Western blot shows Class I compounds dissociate barrier‐forming Nup62 and Nup98 from the NPC (S: supernatant; NE: nuclear envelope), unlike Class II [46]. Schematic (middle, lower) illustrates target sites: Class I disrupts FG‐Nup cohesion; Class II destabilizes Y‐complexes in the scaffold. Adapted and redesigned from previously published experimental observations from our group, with the original studies cited [46].

By pharmacologically overriding the mechanostat, these agents are proposed to exploit the pre‐existing mechanical stress and reduced resilience of the cancer nucleus. In effect, they push the “massive but leaky” nuclear architecture of the cancer cell past its point of mechanical failure—triggering nuclear rupture, DNA damage, and apoptosis while sparing healthy cells that retain robust mechanical buffering capacity.

#### 2.6.1 Class I Compounds: FG‐Domain Dissociation and Mechanical Decoupling

Class I compounds disrupt the hydrophobic interactions among the phenylalanine‐glycine (FG) repeat domains that constitute the NPC's permeability barrier [50, 66]. These intrinsically disordered domains are central to maintaining the spatial confinement and selectivity of the pore. Physically, FG‐domains behave as tethered polymer chains whose configurational entropy and transient receptor interactions generate an entropic barrier to passive diffusion [53, 82]. This “entropic brush model” is highly consistent with our observations of the rapid, reversible NPC dilation that occurs under mechanical and chemical perturbations.

While alternative frameworks—such as the “selective phase/hydrogel model”—propose a cohesive meshwork stabilized by inter‐repeat crosslinks [33], such a structure implies a finite yield stress and slower relaxation dynamics. These features appear less compatible with the near‐instantaneous transport modulation observed in live‐cell imaging [70, 83].

Our findings, particularly the rapid recalibration of nuclear permeability and mechanical response, align more closely with the entropic barrier paradigm. However, we acknowledge hybrid interpretations where FG‐domain behavior may shift toward a cohesive state under specific stress conditions or metabolic states, such as the hyper‐O‐GlcNAcylation observed in malignancy.

Pharmacological dissociation of FG‐domains by Class I agents reduces local polymer density and disrupts entropic exclusion forces, lowering the energy barrier for nonspecific entry while simultaneously increasing central channel compliance. Crucially, FG‐Nups such as Nup153 and Nup62, though distinct from the structural scaffold, are anchored via flexible linkers that transmit force [30]. Their dissociation via Class I agents mechanically decouples the permeability barrier from the scaffold, eroding the NPC's capacity to buffer nuclear stress.

Our AFM‐based indentation studies validate this model: Class I treatment increases overall nuclear deformability and alters the mechanical response of the nucleus under load. These effects mimic force‐induced dilation but are chemically triggered, establishing FG‐domain cohesion as a critical mechanical determinant of nuclear behavior. In cancer cells—which are characterized by elevated NPC density and a pre‐existing reduction in barrier stringency—Class I compounds further destabilize the transport interface. By stripping away the remaining entropic resistance, these agents push the “massive but leaky” cancer nucleus toward its mechanical failure threshold, triggering rupture, and cell death.

#### 2.6.2 Class II Compounds: Scaffold Destabilization and Loss of Elastic Integrity

Class II compounds target structured scaffold proteins that form the NPC's concentric rings [46, 47, 48], specifically members of the Y‐complex (Nup107, Nup133, and Nup160). These adopt β‐propeller and α‐solenoid folds that confer mechanical rigidity and spatial organization necessary for nuclear envelope and NPC stability [35].

In our “nuclear mechanostat” framework, the scaffold serves as a primary load‐bearing frame, distributing cytoskeletal stress and maintaining pore geometry during large‐scale nuclear deformations.

Pharmacological disruption of scaffold integrity—using agents such as Pitstop‐2 and its derivative RVD‐127‐, [84] triggers a loss of ring symmetry and impairs the anchoring of the FG‐domain barrier. From a continuum mechanics perspective, this scaffold failure induces localized stress concentrations across the nuclear envelope. By undermining the structural elasticity of the pore, Class II agents increase the susceptibility to pore collapse and significantly elevate the risk of nuclear envelope rupture during confined migration.

Our experimental data reveal that Class II treatment increases both the frequency of nuclear rupture and overall nuclear deformability under load [46, 84].

These effects are consistent with scaffold failure and mechanical uncoupling from the nuclear lamina, effectively disabling the structural “skeleton” of the NPC. In transformed cells—which critically rely on scaffold flexibility to navigate the dense, high‐stress environments of the tumor microenvironment—this interference targets a vital survival adaptation. By disabling this component of the mechanostat, Class II compounds push the already strained cancer nucleus toward a state of permanent structural failure, leading to genomic instability and cell death.

#### 2.6.3 Cancer Cells as Mechanically Vulnerable Targets: Predicted Selectivity and Required Tumor–Normal Validation

The strategic remodeling of nuclear mechanics in malignancy creates a profound “structural liability” that can be exploited for therapeutic gain. While normal cells maintain a robust lamina architecture and high‐fidelity NPC gating, transformed cells typically operate in a state of chronic mechanophysiological strain. This state is characterized by:

1. *Compromised barrier stringency*: Elevated NPC density combined with metabolic “leakiness” (e.g., hyper‐O‐GlcNAcylation) reduces the energetic threshold for transport [71, 85].
2. *Reduced resilience*: The loss of Lamin A/C—a hallmark of invasive phenotypes—decreases intrinsic nuclear stiffness, rendering the nucleus dependent on the NPC “mechanostat” for structural buffering [7].
3. *Pre‐existing fragility*: Frequent envelope rupture during confined migration already subjects these cells to chronic DNA damage and pro‐inflammatory stress signaling [20].

At present, the preferential sensitivity of malignant cells to Class I and Class II NPC‐targeting compounds should be viewed as a testable prediction derived from their altered nuclear mechanics, rather than as an established pan‐cancer selectivity principle. This prediction is consistent with independent evidence that cancer cells can become selectively vulnerable to perturbation of NPC physiology, including inhibition of NPC formation, whereas normal cells may survive through reversible cell‐cycle arrest [44]. Systematic matched comparisons between malignant cells and non‐transformed counterparts will be essential to define therapeutic windows, tissue specificity, and resistance mechanisms.

These adaptations are a trade‐off: they enable the high‐volume transport and extreme deformability required for invasion, but they simultaneously render the cancer nucleus mechanically fragile [4, 5]. Our work suggests that pharmacological disruption of NPC mechanics—via either Class I FG‐domain decoupling or Class II scaffold destabilization—pushes these cells beyond their mechanical tolerance limit.

By targeting NPC barrier stringency and scaffold integrity, we destabilize a system that is already operating near its mechanical failure threshold. In malignancy, these perturbations act as a “biophysical final straw,” triggering catastrophic nuclear rupture, total loss of transport fidelity, and apoptotic collapse. In contrast, healthy cells are expected to retain an intact, robustly buffered mechanostat, providing them with a “mechanical safety margin” that grants significant resilience to such interventions [9].

The nuclear mechanostat thus provides a mechanophysiologically guided and experimentally testable framework for precision oncology. By integrating biophysical principles into cancer pathophysiology, we move beyond traditional molecular targeting toward a strategy that exploits the fundamental physical limits of the malignant cell.

## 3 Conclusion and Discussion

The nucleus is increasingly recognized as a dynamic, force‐responsive organelle, in which permeability and mechanical resilience are closely linked. NPCs are emerging as active regulators of nuclear mechanotransduction, integrating biophysical stress with changes in transport selectivity and transcriptional adaptation.

In cancer, this system can undergo pathological remodeling to support proliferation, invasion, and survival. At the same time, such remodeling may expose structural and mechanical vulnerabilities. We propose that targeting NPC barrier stringency or scaffold integrity with Class I and Class II NPC‐modulating compounds could compromise the reduced nuclear resilience and transport fidelity of malignant cells. This reframes the nuclear pore not merely as a conduit, but as a potentially druggable mechanotransductive interface. However, this mechanophysiologically guided strategy requires rigorous validation across tumor types, normal‐cell counterparts, and distinct mechanical contexts to define therapeutic windows, resistance mechanisms, and safety boundaries.

Ultimately, the nuclear mechanostat provides a testable conceptual framework for integrating mechanics into cancer pathophysiology, rational drug design, and nanomedicine. In this view, NPCs are not static gateways but dynamic sensors, biophysical buffers, and candidate therapeutic targets. The mechanostat should therefore be regarded as a model with clear experimental and translational implications for precision oncology.

## Author Contributions

S.T.S. conceived the review framework , performed the literature synthesis, and wrote the initial article draft. C.R.M. and A.F.S. contributed to literature analysis, conceptual refinement, and article revision. Y.Q. and W.‐E.Y. contributed to the conceptual development, translational framing, and critical review of the article. V.S. supervised the work, contributed to the central conceptual framework, and critically revised the article. All authors discussed the content, contributed to the final article, and approved it for submission.

## Funding

This study was supported by Deutsche Forschungsgemeinschaft (SH 167/6‐1, SH 167/6‐2, SH 167/9‐1, and SH 167/15‐1) and Sino‐German Mobility Programme (Grant number: M‐0699).

## Conflicts of Interest

The authors declare no conflicts of interest.

## Data Availability

The data that support the findings of this study are available from the corresponding author upon reasonable request.
